# COVID-19 and older adults: more support is needed

**DOI:** 10.1016/j.eclinm.2020.100532

**Published:** 2020-09-03

The COVID-19 pandemic has exposed a litany of inequalities in societal health care, from differences in the impact on racial groups to regional discrepancies. In 2020, no group has been more vulnerable than the older community. Although the immediate concern for this group is mortality, the COVID-19 crisis is likely to have a profound effect on both the mental and physical outlook of these individuals as a result of disruptions to social interactions, contact, and support.

Today, one in every 12 people in the UK is aged older than 75 years, and by 2040, this number is expected to nearly double to one in seven, with similar numbers expected for many high-income countries. Older adults need complex health care, often because of multimorbidities. This increased burden on health-care systems presents a challenge for all and, if not specifically addressed, may cause a crisis. With this in mind, over the past decade there has been an increased focus on addressing these challenges using a variety of approaches. The private sector has geared up investment in healthy longevity programmes focusing on neurodegenerative diseases, artificial intelligence, and apps for medicine compliance and deep learning for disease prediction. Governments recognising both the appeal to older voters and the challenges to health care have also focused on initiatives for the older population. As part of the UK Government's Grand Challenge missions, the focus on Ageing Society aims to ensure that by 2035 the population achieves an extra 5 years of healthy living. The programme, launched in 2019, seeks to support this challenge with more than £200 million in investments and partnerships with local innovative initiatives. The ultimate aim of such campaigns seeks a better standard of living, while ensuring older people are able to work and engage with the rest of society for longer.

More than a third of all visits to doctors in the UK are for non-acute, broad issues, including loneliness and moderate mental health concerns. Social interventions into healthy ageing are, and will continue to be, an essential part of the drive towards more healthy ageing. In practise, this means point of care physicians are increasingly prescribing social interventions to individuals, as appropriate. These interventions fall into a number of categories. Psychological therapies, such as humour therapy, mindfulness, stress-reduction therapy, and reminiscence group therapy can have a positive effect on loneliness and life satisfaction outcome measures. Social facilitation interventions will often involve group meetings in a charity or care provided setting. Friendship enrichment programmes, cultural identity programmes, and computer learning-based solutions all seek to stimulate interactions, movement, and cognitive exercise. The primary physician might action this as a referral, in which case the patient is transferred to link workers, who coordinate and facilitate the community intervention. For primary health-care providers, this intervention directly addresses the issues befalling the patient, which might otherwise not be solved medically and ensures the patient has a direct contact for follow-up. The link worker focuses on tailoring and linking the patient to available interventions in their community or within local reach via charities or organisations such as *NextDoor*. Such programmes are funded by local health-care providers or charitable organisations. Unfortunately, because services differ regionally and locally, regional inequalities can result. Cost-effectiveness of these interventions has been shown over incorrectly offering medicine prescriptions for these individuals, demonstrating a 4:1 cost-saving efficiency.

Novel positive interventions to improve the wellbeing of older adults are essential, as the growing increase in the ageing adult population. In 2019, WHO published “Intersectoral action: the arts, health and well-being”. This call to action followed on from a WHO report showing the ways in which novel art interventions can partner with health care to inform healthy living across various age ranges. The report shows the multiple ways in which regular exposure to arts (community theatre, choirs, painting, and art classes) can improve a number of problems, both acute and chronic, such as stress, cognitive decline, and pain management in older adults while removing stigma around this age group. Social prescription and social intervention, as their names suggest, are supporting social interaction and, as such, COVID-19 has had a devastating effect. The combination of a patient group significantly being more at risk of serious illness, and treatments, based on face-to-face contact has been a challenge for health care and link workers. Health-care workers have sought to mitigate these issues, and tried to address the challenges. The link between primary care physician and link worker has been maintained virtually, with general practitioners referring new inpatients and sharing lists of potentially more vulnerable individuals who require support. Link workers are adapting to their responsibilities via a framework created by the National Association of Link Workers. Telephone and video appointments have been massively upgraded. Centralised Facebook groups of social distancing-appropriate events, such as virtual life drawing sessions and virtual open gardens, have been created. Link workers have also been encouraged to increase coordination with the Voluntary Community and Social Enterprise (VCSE). The VCSE comprises a number of local and national charity groups established to support the voluntary sector. Connection between link workers and the VCSE allows greater access to the most vulnerable across a number of communities via shared resources.

Unfortunately, to date, many of the community programmes that form the basis of the social interventions remain closed. Resistance, hesitancy, and insecurity to technology for many older adults means that this approach might never be the outright solution to COVID-19's challenges. However, as screen-to-screen telecommunication becomes an ever more prominent part of everyday life, integration of the older populations must be increased. Data are beginning to show an increased usage of the internet, mobile phones, and, importantly, smartphones by the over 65s. Charities such as *Age UK* have implemented a free tablet distribution programme to vulnerable and isolated individuals, and have received positive feedback. Collaboration between technology companies and health care must urgently consider this opportunity to assist older adults in their knowledge and understanding of communication technology.

Older adults have borne the brunt of the SARS-CoV-2 pandemic. From increased mortality and morbidity, to the mass mismanagement and treatment of older adults in care homes in the UK, the pandemic has exposed our failure to protect this group. As the long-term impact of this pandemic begins to reveal itself across all ages and groups, we must be vigilant of the impending social crisis caused by the loss of these services to older adults. Besides the emotional bonds we have with older adults, many contribute to society as volunteers and caregivers. Never has it been more important to find innovative ways to protect and support the most at-risk members of society.

*EClinicalMedicine*

